# The Effect of Low-Frequency Road Noise on Driver Sleepiness and Performance

**DOI:** 10.1371/journal.pone.0123835

**Published:** 2015-04-15

**Authors:** Anna Anund, Eva Lahti, Carina Fors, Anders Genell

**Affiliations:** 1 Swedish National Road and Transport Research Institute, Linköping, Sweden; 2 Rehabilitation Medicine, Linköping University, Linköping, Sweden; 3 Volvo Car Cooperation, Gothenburg, Sweden; Kyoto University, JAPAN

## Abstract

It is a well-known fact today that driver sleepiness is a contributory factor in crashes. Factors considered as sleepiness contributor are mostly related to time of the day, hours being awake and hours slept. Factors contributing to active and passive fatigue are mostly focusing on the level of cognitive load. Less is known what role external factors, e.g. type of road, sound/noise, vibrations etc., have on the ability to stay awake both under conditions of sleepiness and under active or passive fatigue. The aim of this moving base driving simulator study with 19 drivers participating in a random order day and night time, was to evaluate the effect of low-frequency road noise on driver sleepiness and performance, including both long-term and short-term effects. The results support to some extent the hypothesis that road-induced interior vehicle sound affects driving performance and driver sleepiness. Increased low-frequency noise helps to reduce speed during both day- and night time driving, but also contributes to increase the number of lane crossings during night time.

## Introduction

It is a well-known fact today that driver sleepiness is a contributory factor in crashes [1,2]. In particular, an increased risk has been reported when driving during the night or early morning [2–5], for young drivers [6,7] as well as for professional drivers [8–10], but also for shift workers driving home after a night shift [11,12] and for people with untreated sleep disorders [8–10,13]. The terms sleepiness and fatigue are often used synonymously, even though the causal factors contributing to the driver state may differ [14]. There might be reason to consider differences between sleepiness and driver fatigue when it comes to the effect of countermeasures. Countermeasures might be either on an operative level (to handle driver fatigue when it occurs) or on a tactical level (to prevent driver fatigue from occurring). Less is known what role external factors, e.g. type of road, sound/noise, vibrations etc., have on the ability to stay awake both under conditions of sleepiness and under active or passive fatigue.

Previous studies have shown that infrasound and low-frequency noise can lead to increased fatigue and also to elevated stress in drivers. Studies have been made on lorry drivers [15], but also in the simulator [16,17] and in laboratories [18], although the sound environment under study in simulator and laboratory was designed to mimic that inside a lorry. The results suggest that low-frequency noise contributes to increased driver fatigue and reduced task performance. The results did not include difference in effects from both a long-term and a short-term perspective and it is not known if there are any carry-over effects.

While the infra- and low-frequency sound levels inside a car cabin are lower than those inside a lorry cabin, the interior sound is still dominated by its low-frequency content since the efficiency of the various insulation, absorption and damping measures employed declines with decreasing frequency [19]. As yet there are no requirements on noise in relation to sleepiness. The aim of this study was to evaluate the effect of low-frequency road noise on driver sleepiness and performance, including both long-term and short-term effects.

## Method

### Participants

A random sample of 20 drivers (30–50 years old) with self-reported good hearing capacity was recruited. One man withdrew prior to the experiment and in total there were 9 men with an average age of 39.4 years (sd 7.3) and 10 women with an average age of 41 years (sd 8.8). Before arrival they were asked to prepare for the preceding 72 hours in order to avoid confusion with other issues. The preparations included abstention from alcohol for 72 hours before the experiment, keeping sleep diaries, at least 7 hours of sleep during the last three nights before arrival, going to sleep no later than midnight and not getting up before 6h. The drivers’ average Epworth sleepiness scale [20] (ESS) score was 7.2 (sd 2.3) for men and 9.3 (sd 3.9) for women. A total score of less than 10 are regarded as normal alert, but there was one woman who scored 18, one who scored 13 and one scoring 12, values that may be considered rather high. The participants drove twice: one session during the daytime/evening (alert) and one session during night time (sleepy). The order was balanced. The participants arrived 2 hours before the experiment started. After the day session they went home by themselves. Before and after the night session they arrived at the laboratory and returned home again after the test by taxi. The participants were paid 3,000 SEK for their participation. VTI has performed simulator experiments with sleepy drivers repeatedly the last 10 years. In line with the Helsinki declaration and the Swedish ethical constitution all participants signed informed consent before the start. All work follow the *Ethical approval* received from the regional ethical committee (Regional Etik Prövnings Nämnd, EPN) in Linköping, Sweden (Dnr 03–376).

### Platform

**Fig 1:**
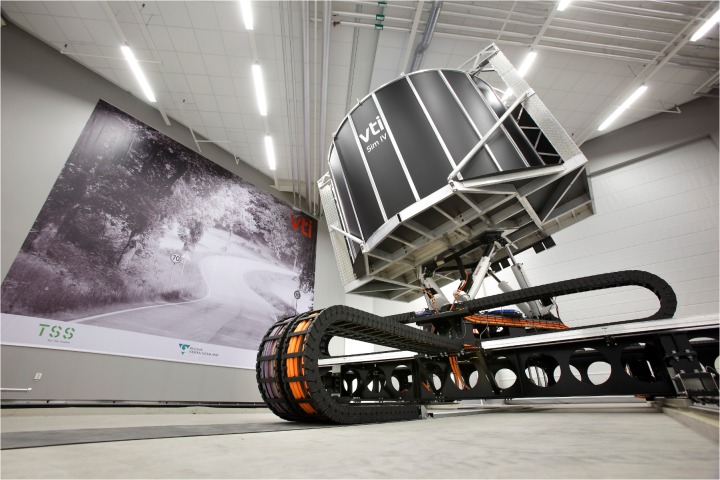
VTI’s simulator IV.

VTI's Driving Simulator IV (Sim IV) was used for the experiment (Fig 1). The simulator has a 180 degree forward field of view and three LCD-displays for rear view mirrors. Sound is presented through a custom-built 6.1 surround sound system. For the current experiment the simulator was equipped with a Volvo XC60 car cabin and the scenario implemented an automatic gearbox.

### Simulation of the sound

The sound in the simulator was modelled after recordings in real vehicles in combination with texture measurements of real road surfaces in order to create conditions as realistic as possible, see also ISO standards 5128:1980 (http://www.iso.org/iso/home/store/catalogue_tc/catalogue_detail.htm?csnumber=11127). The two driving conditions were represented by either a vehicle of large wagon type driven on a relatively smooth asphalt road surface (quiet case), or a vehicle of mid-size hatchback type driven on a relatively coarse concrete road surface (loud case). As the interior vehicle sound is low-frequency dominated, both the difference between dB(A) and dB(C) levels, as well as G-weighted levels were used in addition to the generally adopted dB(A) level to quantify the low-frequency content of the sound environment [21]. The absolute levels of the vehicles measured differed slightly from the levels in the simulator, but the difference between the two experimental conditions was similar to the difference recorded in the real vehicles; see Table 1. The sound shows similar differences between A-weighted and C-weighted sound pressure levels, indicating that all sounds have a similar low-frequency emphasis. A difference of 15dB between A-weighted and C-weighted levels is the Swedish occupational health legislation limit for when specific low-frequency related measures are to be taken into account.

**Table 1:** A and C-weighed levels of the measured and corresponding modelled sound of the different conditions.

Method	Sound	dB(A)	dB(C)	Diff A	Diff C-A
Measured	A (quite case)	79.8	94.4		14.6
	B (loud case)	84.8	99.9	5.0	15.1
Modelled	A (quite case)	82.6	97.7		15.1
	B (loud case)	86.7	102.9	4.1	16.2

Previous studies of noise-induced driver fatigue have mainly focussed on heavy vehicles and their relatively prevalent high levels of infrasonic frequencies. In the current study the focus was on the differences in the sound due to properties of the road surface and due to differences between two sizes of car models (large and mid-size). When investigating the infrasonic content of the recorded interior sound for these conditions, no major difference was found between the conditions, apart from a slight increase in levels for frequencies around 8Hz, see

**Fig 2:**
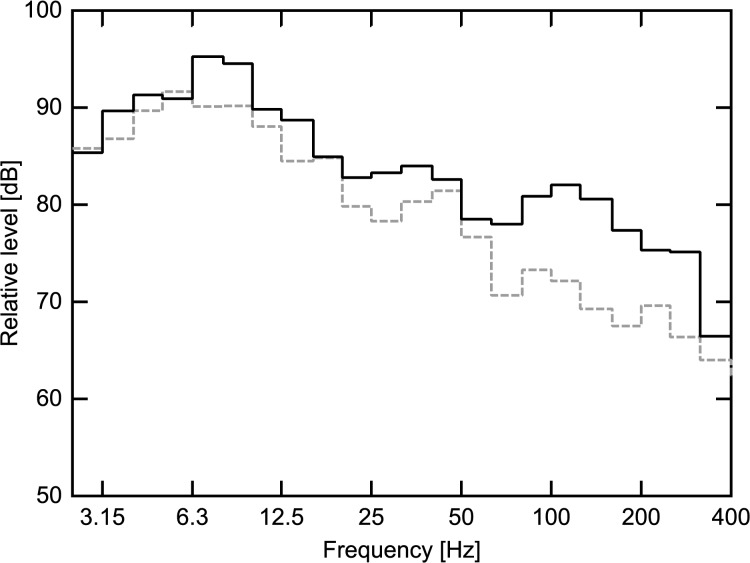
Infrasonic spectra of recorded vehicle sounds in 1/3 octave bands.

**Fig 2**. This difference can also be found in the G-weighted sound pressure levels which are sensitive to differences in infrasonic frequencies. The large wagon has a G-weighted sound pressure level of 85.8 dB(G) and the mid-sized hatchback a level of 88.6 dB(G). The sound in the simulator was filtered so that frequencies below 12Hz were removed, thereby removing the influence of this infrasonic difference and its unwanted influence in the study. The resulting two conditions used in the driver sleepiness experiment were evaluated by a panel of vehicle noise experts who agreed on the validity of the sounds. The evaluation was done as a workshop were the different sounds were rated subjectively and then discussed.

### Design and procedure

Two auditory stimuli were used;

- quiet case: large wagon driving on a relatively smooth asphalt road surface.
- loud case: mid-sized hatchback driving on a relatively coarse concrete road surface

**Fig 3:**
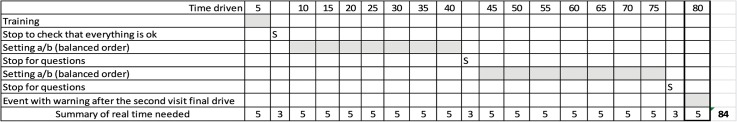
The “protocol” for the driver.

The order between A (quiet case) and B (loud case) was balanced between participants, but it was the same for daytime and night-time conditions. Each session started with 5 minutes’ training followed by a 3-minute break and then 35 (first case) + 35 (second case) minutes of driving (**Fig 3**). The training used the same case setting (A or B) as the first setting in the following part. Between each 35-minute drive there was an intermission where the participants were asked questions while they remained seated in the simulator. The vehicle was placed at the beginning of the road after the break. After the final 35 minutes, at the last visit, the participants kept on driving for approximately 5 minutes more while performing a task aimed at testing the effectiveness of a warning sound when leaving the lane unintentionally. These data have not been used here.

There were two participants each day and each night. The first daytime participant arrived at 15h for preparation and drove, presumed alert, between 16-18h. The second participant arrived at 17h and drove, presumed alert, between 18-20h. The first night-time participant arrived at 00h and drove, presumed sleepy, between 02-04h. The second participant arrived at 02h and drove, presumed sleepy, between 04-06h. The night-time participants came to and left the laboratory by taxi.

### Scenario

The simulated scenario was a motorway with a speed limit of 110 km/h, daylight and a small amount of fog. The road was based on the 40 km E4-simulation. There was no traffic in the same lane as the driver, but some oncoming vehicles to make the scenario more realistic. There were milled rumble strips at the shoulder. These were presented visually and if the drivers crossed them a sound from the rumble strips was activated. The rumble strips were intended to support the drivers in case they fell asleep and to wake them up so as to avoid simulator stoppage. The drivers did not know the aim of the study, nor did they know that some of the experimenters represented VCC.

### Measures

The simulator data acquisition system was used to sample speed, lateral position and steering wheel angle. Data were sampled at a frequency of 50 Hz. Blink behaviour (EOG) and heart rate (ECG) were recorded by a Vitaport 3 (TEMEC Instrument B.V., The Netherlands) with a sampling frequency of 512 Hz for EOG and 256 Hz for ECG. Blink parameters were calculated by the LAAS algorithm [22]. Vertical EOG from the right eye were used as input data to the algorithm. The participants reported their subjective sleepiness by using the Karolinska Sleepiness Scale as an average once every 5 minutes [23]. Questions to the participants were asked before the experiment started, after each 35 minutes’ driving and after the finalisation of each experiment. A scale from 1 (not at all) to 7 (very much) was used and the drivers rated if they felt or experienced that the drive had been demanding, boring, worried, irritating, monotonous, motion-sickness-inducing, realistic. After the second drive they were asked if they found any differences between the first and the second drives.

### Data analysis

In total 20 drivers were recruited, but due to sickness one did not arrive as planned and hence 19 drivers participated in the study. The total dataset thus consists of 19 participants x 2 (day vs. night) x 2 (quiet case vs. loud case) x 7 (segments per condition) = 532 segments. Out of the 532 segments, 32 were excluded for various technical and practical reasons.

Six performance indicators (PI) were used in order to analyse driving performance and sleepiness level: Karolinska Sleepiness Scale (KSS) [24], blink duration (s), mean speed (km/h), average and variability of lateral position (m)—measured relative to the centre of the road, average number of lane crossings to the left or to the right per km driven. All PI were calculated in time windows, called segments, which corresponded to the 5-minute time intervals for which KSS were reported. However, in order to avoid including the acceleration phase in the beginning of each session, the first 20 s of all 5-minute windows were excluded. Thus, all driving and physiological data were calculated in time windows that started 4:30 before the KSS text appeared and ended when the KSS text disappeared (10 s after appearance). In total, there were 7 segments per 35 minutes driving.

Initially, a possible carry-over effect was tested for with the aid of a Mixed Model Anova. The model used Subject as random and fixed factors for condition (day/night), minutes driven (5–35) and sound (quiet/loud) and also a factor for first/second part of the drive. The model included main effects and interaction on first, second and third levels.

An analysis was also done for a reduced data set containing only the first part (35 minutes) of each driving session (day or night), to evaluate the differences between the “loud” and “quiet” without the influence of a possible carry-over effect. Here as well, a mixed Model Anova was used with subject as random and fixed factors for day/night, minutes driven (5–35) and sound (quiet/loud). The model included main effects and interaction on first level and second level. Influences of “loud” or “quiet” sound settings on responses to the questionnaires were analysed using t-tests (alpha = 0.05). All analysis was done using SPSS version 19.0.

## Results

### Performance Indicators

**Fig 4:**
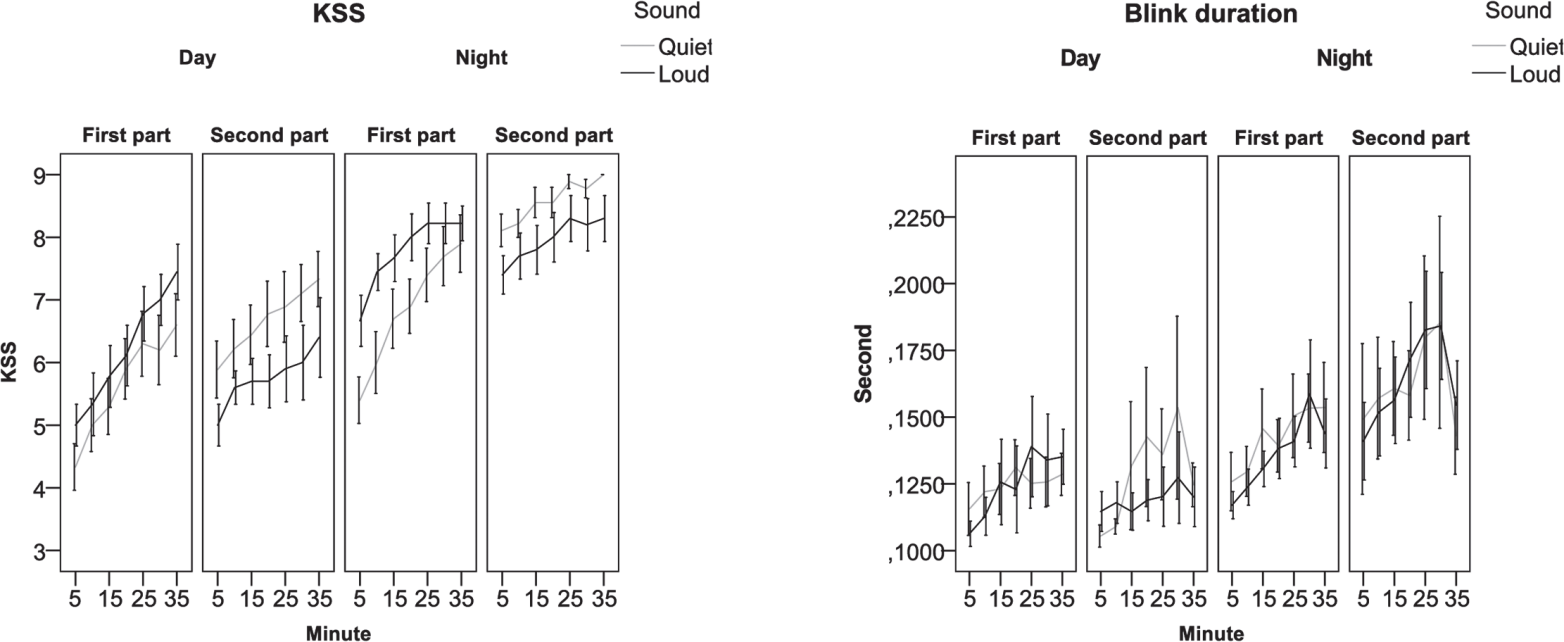
Sleepiness indicators related to subjective sleepiness and blink behaviour divided by Condition (day and night); Case setting (quiet and loud) and First/second part of the drive and time on task (5-10-15-20-25-30-35 minutes).

**Fig 5:**
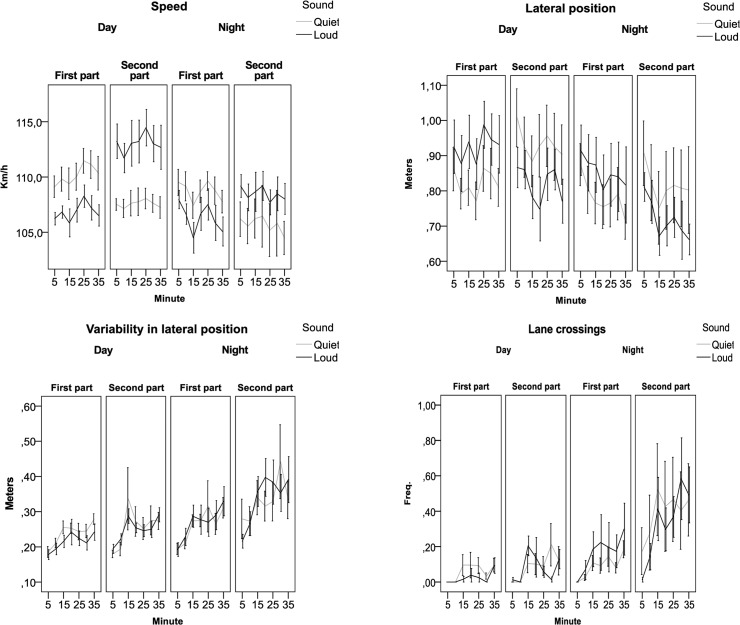
Performance indicators related to speed and lateral position divided by Condition (day and night); Case setting (quiet and loud) and first/second part of the drive and time on task (5-10-15-20-25-30-35 minutes).

For the full data set, there were no significant main effects of Sound for KSS, Blink duration, Speed, Lateral position (mean and variability) or Lane crossings; see Fig 4 and Fig 5. There were significant effects for Condition (day/night) and Part of the drive (first/second) for all variables. This was also true of Time on task (Minute) except for Speed. The results for KSS and Speed show that after the break the drivers continued to behave as they had done just before the stop, regardless the new type of sound. This means that the result supports a lasting effect of a specific sound (quiet or loud) even if the driver changed to another car—a so-called carry-over effect; see Table 2. There were interactions (two-level) between Condition and Part of the drive for all variables. This might be interpreted as if there is a different effect on the participant’s comparing the first and the second part of the drive, daytime compared to night time. In addition there was a three-level interaction between Condition*Part*Sound for Blink duration, and for Speed.

**Table 2:** Mixed Model Anova: condition (day/night); Part (first/last); Minute (0–35); Sound (quiet/loud).

	KSS	Blink dur.	Speed	LP	SDLP	LC
**Cond. (day/night)**	**345.555 (p<0.01)**	**52.209 (p<0.01)**	**52.667 (p<0.01)**	**109.466 (p<0.01)**	**53.136 (p<0.01)**	**60.435 (p<0.01)**
**Part (First/Last)**	**44.223 (p<0.01)**	**19.568 (p<0.01)**	**5.636 (p<0.01)**	**10.722 (p<0.01)**	**32.144 (p<0.01)**	**35.884 (p<0.01)**
**Minute (5–35 min)**	**23.143 (p<0.01)**	**7.573 (p<0.01)**	1.612 (p = 0.14)	**10.599 (p<0.01)**	**13.259 (p<0.01)**	**6.903 (p<0.01)**
**Sound (quiet/loud)**	0.043 (p = 0.84)	1.012 (p = 0.31)	3.407 (p = 0.07)	2.933 (p = 0.09)	0.197 (p = 0.66)	0.010 (p = 0.92)
Cond. * Part	**13.400 (p<0.01)**	**11.882 (p<0.01)**	**16.490 (p<0.01)**	**8.389 (p<0.01)**	**6.310 (p<0.01)**	**16.732 (p<0.01)**
Cond. * Minute	0.370 (p = 0.90)	0.499 (p = 0.81)	0.836 (p = 0.54)	1.999 (p = 0.07)	1.166 (p = 0.32)	1.361 (p = 0.23)
Cond. * Sound	3.151 (p = 0.08)	0.079 (p = 0.78)	0.984 (p = 0.32)	0.066 (p = 0.80)	1.526 (p = 0.22)	0.140 (p = 0.71)
Part * Minute	**2.644 (p = 0.02)**	0.961 (p = 0.45)	1.287 (p = 0.26)	1.455 (p = 0.19)	0.717 (p = 0.64)	1.014 (p = 0.41)
Part * Sound	**4.798 (p = 0.04)**	0.097 (p = 0.76)	**5.866 (p = 0.02)**	1.319 (p = 0.27)	0.018 (p = 0.89)	0.070 p = 0.79)
Minute * Sound	0.173 (p = 0.98)	0.295 (p = 0.94)	0.187 (p = 0.98)	0.734 (p = 0.62)	0.597 (p = 0.73)	0.242 (p = 0.96)
Cond.* Part * Minute	0.166 (p = 0.99)	0.455 (p = 0.84)	0.382 (p = 0.89)	0.914 (p = 0.48)	0.394 (p = 0.88)	0.265 (p = 0.95)
Cond.* Part * Sound	0.071 (p = 0.79)	**14.876 (p<0.01)**	**12.814 (p<0.01)**	0.179 (p = 0.67)	0.154 (p = 0.69)	2.039 (p = 0.15)
Cond.* Minute* Sound	0.346 (p = 0.91)	0.522 (p = 0.79)	0.088 (p = 0.99)	0.269 (p = 0.95)	0.540 (p = 0.78)	0.847 (p = 0.53)
Part* Minute* Sound	0.058 (p = 0.99)	0.278 (p = 0.95)	0.158 (p = 0.98)	0.811 (p = 0.56)	0.837 (p = 0.54)	0.044 (p = 0.99)

F-values bold show significance. P-values in parenthesis.

Since the results of the data set showed the presence of a carry-over effect, the influence of low-frequency road noise on sleepiness was analysed by taking into account only the first 35 minutes driven during both day and night time. The results did not show any significant main effects of sound either on the sleepiness indicators or the driving related indicators, except for Speed. The drivers drove faster in the quiet case than in the loud case during daytime (110 km/h in quiet case vs 106 km/h in loud case) and during night time (109 km/h in quiet case vs 106 km/h in loud case).

There were significant interactions between Condition*Sound for Blink duration (F = 5.01; p = 0.03) and for Lane Crossings (F = 6.35;p = 0.01) indicating that the effect of sound differs depending on daytime or night-time driving. On average, blink duration during daytime was 0.131s (sd 0.01) for the loud case compared to 0.125s (sd 0.01) for the quiet case. During night time the average blink duration was 0.145s (sd 0.01) for the quiet case compared to 0.139s (sd 0.01) for the loud case. However, even if these differences are significant, the absolute difference is small and based on few people’s contribution.

**Fig 6:**
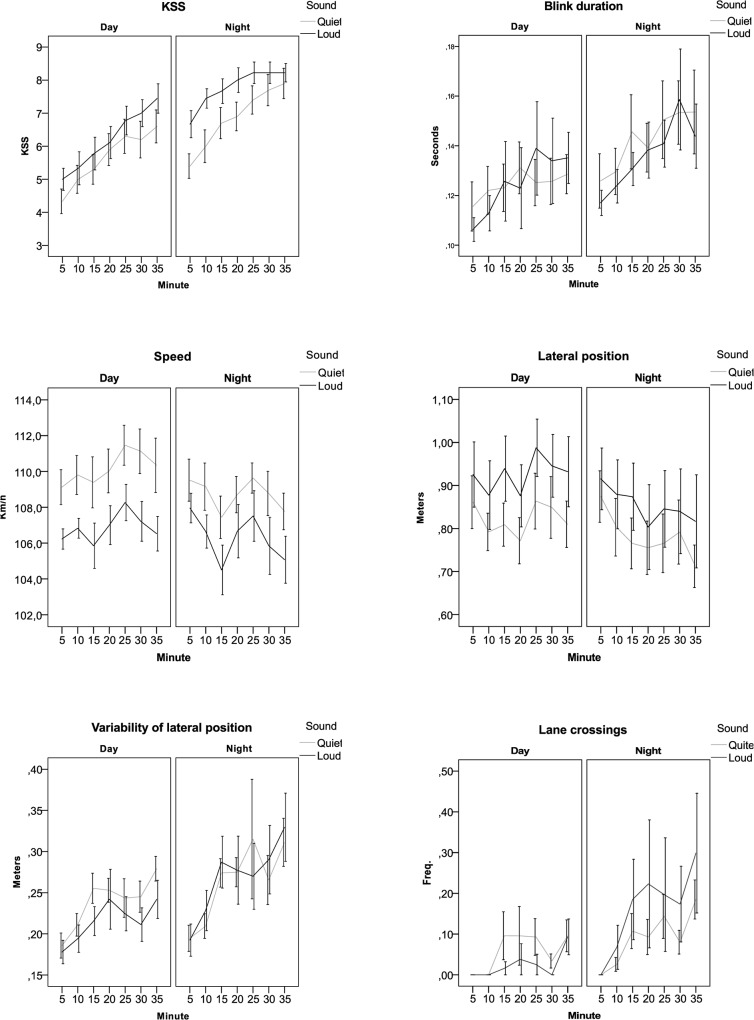
Sleepiness and performance indicators for the first 35-minute drive divided by condition (Day and Night) as function of time on task (5-10-15-20-25-30-35 minutes) and type of sound (Quiet or Loud).

On average, the quiet case generated more lane crossings during daytime driving than the loud case (0.058; sd 0.05 vs. 0.030; sd 0.04). During night time the opposite applied, with more lane crossings during the loud case (0.165; sd 0.04) than during the quiet case (0.096; 0.04). It should be kept in mind that the number of lane departures remained small. For all variables there were significant differences between day and night-time driving as well as regarding how long the driver had been driving, i.e. “time on task”. There were major differences between individuals; see Table 3 and **Fig 6**.

**Table 3:** Mixed Model Anova: condition (day/night); minute (0–35); sound (Quiet/Loud).

	Cond. (day/night)	Minute (5–35 min)	Sound (quiet/loud)	Cond. *Minute	Cond. *Sound	Minute *Sound	Cond. *Minute *Sound	Partic. (Wald Z)
**KSS**	**157.5 (p<0.01) (df 1. 220)**	**29.0 (p<0.01) (df 6. 220)**	2.40 (p = 0.14) (df 1. 17)	0.46 (p = 0.84) (df 6. 220)	3.03 (p = 0.08) (df 1. 220)	0.21 (p = 0.97) (df 6. 220)	1.05 (p = 0.39) (df 6. 220)	**2.76 (p<0.01)**
**Blink dur.**	**24.78 (p<0.01) (df 1. 204)**	**7.94 (p<0.01) (df 6. 203)**	0.00 (p = 0.99) (df 1. 17)	0.90 (p<0.49) (df 6. 202)	**5.01 (p = 0.03) (df 1. 203)**	0.84 (p<0.54) (df 6. 204)	0.84 (p<0.54) (df 6. 202)	**2.77 (p<0.01)**
**Speed**	**7.78 (p<0.01) (df 1. 211)**	**3.26 (p<0.01) (df 6. 209)**	**6.35 (p = 0.02) (df 1. 17)**	1.1 (p = 0.36) (df 6. 209)	3.54 (p = 0.06) (df 1. 211)	0.21 (p = 0.97) (df 6. 211)	0.06 (p = 0.99) (df 6. 209)	**2.66 (p<0.01)**
**LP**	**46.33 (p<0.01) (df 1. 209)**	**6.71 (p<0.01) (df 6. 209)**	1.04 (p = 0.32) (df 1. 17)	**3.88 (p<0.01) (df 6. 209)**	0.16 (p = 0.69) (df 1. 209)	0.97 (p = 0.56) (df 6. 209)	0.23 (p = 0.97) (df 6. 209)	**2.89 (p<0.01)**
**SDLP**	**20.27 (p<0.01) (df 1. 211)**	**10.34 (p<0.01) (df 6. 209)**	0.10 (p = 0.76) (df 1.17)	0.85 (p = 0.54) (df 6.209)	3.04 (p = 0.08) (df 1. 211)	0.26 (p = 0.96) (df 6. 209)	0.51 (p = 0.80) (df 6. 209)	**2.58 (p<0.01)**
**Lane Crossings**	**19.90 (p<0.01) (df 1. 211)**	**5.49 (p<0.01) (df 6. 209)**	0.16 (p = 0.69) (df 1.17)	0.91 (p = 0.49) (df 6.209)	**6.35 (p = 0.01) (df 1. 211)**	0.18 (p = 0.98) (df 6. 209)	0.41 (p = 0.87) (df 6. 209)	**2.50 (p<0.01)**

F-values bold show significance. P-values and df in parenthesis.

### Questionnaire

The drivers found the driving rather monotonous, boring and demanding. They were not worried or irritated, regardless of condition, part of the drive or type of noise, but professed to be more worried during night time driving compared to daytime (t = -3.26;p<0.01) They found it more demanding to drive during night time compared to during daytime (t = -4.77;p<0.01). There were no other significant differences between the drivers’ answers comparing daytime and night-time driving, quiet or loud case or first or second part of the drive. After the second part of the drive, both daytime and night-time, the drivers were asked to compare the second and the first parts of the drive. They found the second and the first parts of the drive very similar, and there was no significant difference between day time (6.0; sd1.6) and night time (6.3; sd1.0) experience.

## Discussion

The results from this study support to some extent the hypothesis that road-induced interior vehicle sound affects driving performance and driver sleepiness. Increased low-frequency noise helps to reduce speed during both day- and night time driving, but also contributes to increase the number of lane crossings during night time.

The effect on indicators of sleepiness and driving, comparing daytime and night-time driving is in line with earlier research in simulators [6,25]. Speed decreases, placement is closer to the centre of the road, and the variability of lateral position increases, with a consequent rise in the frequency of lane crossings. Self-reported sleepiness KSS and blink duration also increase in the same way as normal in such studies.

Barring major individual differences, night-time blink duration seems to increase more during the quite case compare to the loud case. The reason for this is not known, but might be related to differences between the more task-related fatigue occurring in daytime and the more sleepiness-related fatigue during night time. Even though there is no significant interaction between Condition*Sound on Speed, there is a clear tendency towards such an effect (p = 0.06). This too might indicate that there are different mechanisms involved, e.g. fatigue versus sleepiness, and that sound affects these mechanisms differently. This is in line with what has been suggested earlier in relation to countermeasures to driver fatigue [14]. This interpretation is also supported by the KSS, with a clear tendency (p = 0.08) for a greater influence of the loud case during night time compare to daytime, with highest KSS at night time for the loud case. In conclusion, further investigations are needed in order to understand the effect of sound on driver sleepiness. That effect might be more U-shaped than linear, meaning that both quiet and loud environments contributes to fatigue and sleepiness. Research is also needed in order to understand why the sleepiness indicators point in different directions.

The drivers drove more slowly during the “loud” sound sessions. This corroborates the proposition of sound being a cue for speed perception, as has been found in previous studies [26]. But it might also argue in favour of impaired driving performance, since earlier studies have shown speed reduction to be one of the sensitive indictors of driver sleepiness [25].

The sound settings used in this experiment were closely modelled after real conditions, albeit specifically severe in the “loud” case in terms of road surface texture [27]. The participants were not informed beforehand about the difference in settings, and they did not notice any difference between the conditions when asked to compare them at the end of the last visit. Thus, the effects from the study can be considered as an involuntary reaction, rather than a result of a conscious reaction. As a consequence, the effects on driver performance found in the experiment are likely to also be valid for real driving, but here again, further studies are needed.

When driving on real roads, the road-induced interior vehicle sound influencing the driver is a combined effect of the type of surface and the silencing of the car. From a countermeasure point of view a selection of “quiet” surface will contribute not only to less in-vehicle noise, but also to a reduction in external noise that may also benefit those living close to the road. A change of road surface is, however, an expensive countermeasure that takes years to implement. If the aim is to reduce, as fast as possible, the contribution of noise to sleepiness requirements, in-vehicle noise is a faster way to reach an effect. Further experiments are then needed, taking into account different levels of speed, different types of road surface and their combinations.

There are several limitations to discuss. One of the major ones relates to the design. A carry-over effect was found, suggesting that the initial condition of each driving session dominated the responses for the entire session. This implies not only a need for methodological considerations, but also for some insight into behavioural aspects. For the methodological part, the two conditions of each session need to be separated by more than just a short questionnaire session while still seated in the simulator, in order to return the participants to their initial state. Considering that there might be a difference in the effect of a countermeasure like a short break, daytime compared to night time [14], there might be reason to consider another type of design for an experiment like this, since it has been proven that the only long-term effective night-time countermeasure is sleep [28]. The results suggest that drivers may take a long time changing their state of sleepiness or fatigue where the environment has been a contributory factor, so that even if the environment changes for the better, the influence of previous worse environment indicate a lasting effect.

## Supporting Information

S1 File Supporting Information. (XLSX)
